# Amino Acids and Hypertension in Adults

**DOI:** 10.3390/nu11071459

**Published:** 2019-06-27

**Authors:** Eleonora Poggiogalle, Mario Fontana, Anna Maria Giusti, Alessandro Pinto, Gino Iannucci, Andrea Lenzi, Lorenzo Maria Donini

**Affiliations:** 1 Department of Experimental Medicine-Medical Pathophysiology, Food Science and Endocrinology Section; Sapienza University of Rome, 00185 Rome, Italy; 2 Department of Biochemical Sciences “A. Rossi-Fanelli”; Sapienza University of Rome, Piazzale Aldo Moro 5, 00185 Rome, Italy; 3 Department of Internal Medicine and Medical Specialties, Sapienza University of Rome, Viale del Policlinico 155, 00165 Rome, Italy

**Keywords:** amino acids, blood pressure, humans

## Abstract

Accumulating evidence suggests a potential role of dietary protein among nutritional factors interfering with the regulation of blood pressure. Dietary protein source (plant versus animal protein), and especially, protein composition in terms of amino acids has been postulated to interfere with mechanisms underlying the development of hypertension. Recently, mounting interest has been directed at amino acids in hypertension focusing on habitual dietary intake and their circulating levels regardless of single amino acid dietary supplementation. The aim of the present review was to summarize epidemiological evidence concerning the connection between amino acids and hypertension. Due to the large variability in methodologies used for assessing amino acid levels and heterogeneity in the results obtained, it was not possible to draw robust conclusions. Indeed, some classes of amino acids or individual amino acids showed non-causative association with blood pressure as well as the incidence of hypertension, but the evidence was far from being conclusive. Further research should be prompted for a thorough understanding of amino acid effects and synergistic actions of different amino acid classes on blood pressure regulation.

## 1. Introduction

According to recent epidemiological projections, the global burden of hypertension is associated with a remarkable increase in poor health outcomes. The global age-standardized prevalence of elevated blood pressure was 24.1% in men and 20.1% in women in 2015 [1]. The overall global prevalence of hypertension is expected to increase by 15–20% by 2025. Based on the Global Burden of Disease report, in 2015 a 1.4 fold increase was detected in mortality and disability-adjusted life years (DALYs) due to the presence of elevated systolic blood pressure since 1990 [2]. 

The association between hypertension and dietary and lifestyle factors is well established, with excess weight, lack of sufficient physical activity, and high sodium intake acknowledged as the main contributors [3]. Nonetheless, dietary interventions play a pivotal role in the extant treatment strategies for hypertension [4]. 

Growing interest has been directed to the potential effect of specific nutrients on blood pressure. Association between dietary protein and hypertension has been described, with a high-protein diet exerting beneficial effects on blood pressure [5]; however, the inverse relationship between dietary protein intake and blood pressure described in short-term studies appears to be weakened when considering longer study duration [6]. One potential mechanism for anti-hypertensive effects connected to elevated protein intake may be represented by the replacement of other macronutrients, mainly carbohydrates: if hypotensive effects are attributable to increased protein intake per se or to the concomitant reduction in the proportions of fat and carbohydrate is still unclear [6]. 

Data regarding the potential effect of the type (plant versus animal source) of dietary protein and blood pressure are not consistent [7]. Evidence from observational studies suggesting a favorable effect on blood pressure due to the vegetable protein intake is not further supported by a meta-analysis including data from forty randomized controlled studies [8], with no differences on blood pressure levels related to dietary protein source. Different protein amino acid compositions, and thus, amino acid intake could be the reason for such discrepant results. It is the case of soy protein, a largely investigated source of vegetable protein, in which some amino acids with antihypertensive properties are abundant (i.e., arginine, and cysteine) [9]. Furthermore, amino acids as part of bioactive peptides derived from food proteins may also be relevant in blood pressure regulation through the inhibition of angiotensin converting enzymes [10]. In recent years, several studies have focused on amino acids and hypertension, with conclusive evidence yet to be established. 

The aim of the present review is to summarize epidemiological evidence concerning the connection between amino acids and hypertension. 

## 2. Methods

Relevant peer-reviewed journal articles published in English were identified in the MEDLINE database (the last search was conducted on 31 January 2019); different combinations of the following search terms were used: “amino acids”, “hypertension”, and “blood pressure”. Bibliographic references from eligible articles were reviewed for selection of any additional studies.

Also, the following exclusion criteria were used: any articles concerning secondary hypertension or central hypertension; any paper evaluating inherited disorders of amino acid metabolism; any study regarding hepatic, renal, or musculoskeletal diseases; any article based on amino acid supplementation; any study assessing amino acid derivatives; any study carried out in children and adolescents; and any study conducted in animals. Review articles, letters in response to published articles, editorials, commentaries, and conference abstracts were excluded.

## 3. Results

A total of seventeen studies were included. Study characteristics, study participants, amino acids investigated, and main outcomes are summarized in Table 1. Ten out of seventeen studies evaluated dietary amino acid intake based on different dietary recall methods (e.g., food-frequency questionnaire, 3 day food diary, 4 day dietary record, 24 h dietary recall, and cross-check dietary history method). In six studies, either plasma or serum amino acid concentrations were assessed, and just one study considered urinary amino acids. In one study, the levels of amino acids in the cerebrospinal fluid were also assessed. Three studies relied on principal component analysis. A marked variability was observed in terms of the number and groups of amino acids investigated, varying from one single amino acid to the calculation of a ratio among different amino acids. Ten out of seventeen studies were cross-sectional, six studies had a prospective design (though in the study by Venho et al. [11], only data at the baseline were considered), and in one study, both the cross-sectional and the prospective analyses were performed [12]. In longitudinal studies, the longest duration of follow-up was 10 years. 

## 4. Discussion 

The present review article provides evidence concerning the connections between amino acids and hypertension, focusing on associations between either blood pressure levels or risk of hypertension, and dietary amino acids or amino acid levels in biological fluids. 

Only the study by Stamler et al. [20] reported a favorable relationship between dietary glutamic acid and blood pressure, showing that an elevated dietary intake of glutamic acid was associated with lower values of both systolic and diastolic blood pressure. This finding is not in accordance with results from a Dutch study describing no association between dietary glutamic acid either blood pressure levels or incidence of hypertension [12]. Vegetable proteins are especially rich in glutamic acids. Glutamate is included in the glutathione molecule, with potential antioxidant effects improving blood pressure homeostasis [28]. Ethnicity-related differences in dietary sources of glutamic acid may explain those conflicting observations: in the INTERMAP study the association between glutamic acid and reduced blood pressure was ascribed to the elevated presence of glutamic acid in vegetable protein, in line with prior evidence indicating a lowering effect of plant protein on blood pressure [20]. Nonetheless, a more recent meta-analysis by Rebholz et al. [8] did not support a different association of animal versus plant protein with blood pressure. 

Regarding tyrosine, results are conflicting: in the Rotterdam study [12], a cross-sectional analysis revealed that a high dietary intake of tyrosine was related to lower systolic blood pressure (though results were statistically just marginally significant when considering quartiles of tyrosine intake, but they become statistically significant when considering tyrosine intake as a continuous variable), but no relationship emerged between dietary tyrosine and diastolic blood pressure. However, no association with the incidence of hypertension was described in findings from prospective analysis after a 6 year follow-up. 

Tyrosine effects on hypertension were also evaluated in another study investigating all aromatic amino acids [24] in an Iranian adult study cohort: dietary tyrosine, as well as dietary tryptophan, did not show any association with incident hypertension after a 3.1 year follow-up. Just phenylalanine intake in the highest quartile was associated to significant increased risk of hypertension compared to the lowest quartile. Nonetheless, when considered globally, high total dietary aromatic amino acid intake exhibited a positive significant association with increased risk of incident hypertension [19,22,26,27]. In a Polish observational study assessing plasma amino acid levels, principal component analysis identified phenylalanine in a separated cluster from tyrosine and tryptophan [19]. Tyrosine acts as a precursor for norepinephrine synthesis, therefore, it can modulate norepinephrine levels and affect the sympathetic tone on the vasculature. Findings from animal studies revealed that tyrosine administration in rats lowered blood pressure through central catecholamine action on alpha-receptors [29,30]. However, data from rodents were not confirmed in hypertensive adults undergoing chronic dietary tyrosine supplementation [31]. The majority of phenylalanine is converted to tyrosine, and effects on blood pressure are potentially due to the changes in tyrosine levels. However, phenylalanine *per se* can interfere with tetrahydrobiopterin (BH4) production, a cofactor for aromatic amino acid hydroxylation, involved in the relaxation of the endothelium [32]. In the presence of the high availability of aromatic amino acids, the oxidation of BH4 may result in alterations of its vasoactive properties with detrimental effects on the endothelium [33]. Tryptophan is a precursor for the synthesis of serotonin (5-hydroxytryptamine, 5-HT), a monoaminergic neurotransmitter. Serotonin receptors are present on adrenergic nerves at the level of the sympathetic–vascular junction, potentially explaining the mechanism underlying the influence of 5-HT on the vascular tone [34]. Administration of l-tryptophan induced a reduction in blood pressure in animals [35], and analogous short-term effects were described in hypertensive patients but not in normotensive controls [36]. However, derangements of 5-HT metabolism have been shown in hypertensive patients, and in the long-term, tryptophan effects on blood pressure are unclear. These reasons may explain the lack of association of habitual dietary intake or circulating tryptophan with blood pressure related outcomes [37]. Moreover, tryptophan-containing peptides obtained from enzymatic hydrolysis of food protein have been shown to interfere with the renin–angiotensin axis inhibiting angiotensin-converting enzymes, though further evidence from human studies is needed [38]. Plasma phenylalanine, together with branched chain amino acids (BCAAs) in the same cluster, showed a positive association with both systolic and diastolic blood pressure, whereas no association was found with blood pressure when the other cluster, including the two remaining aromatic amino acids, was taken into account. 

Dietary BCAAs clustered with aromatic amino acids (AAAs), and proline based on principal component analysis in a longitudinal study conducted in an Iranian cohort, showed a positive association with incidence of hypertension [23]. Analogous findings of a positive relationship between BCAAs and AAAs emerged in two Asian studies [26,27]. In the TwinUK cohort, dietary BCAAs were associated with decreased risk of hypertension [15], whereas no relationship was observed in a Chinese study considering serum BCAA levels [14]. In one study taking into account the ratio between different dietary amino acids, the leucine–serine/threonine–tryptophan ratio results were significantly positively associated with the risk of hypertension [22].

Branched chain amino acid concentrations in biological fluids (serum/plasma) were positively associated with high blood pressure, though data were discrepant in some studies based on dietary records. Plenty of studies indicated that alterations in BCAA metabolism, leading to the accumulation of BCAAs and their byproducts, are linked to remarkable metabolic derangements such as insulin resistance, the latter being associated with increased risk of hypertension [39,40]. Furthermore, BCAA ingestion was able to reduce brain tryptophan and tyrosine uptake, affecting serotonin and catecholamine synthesis with consequences on central blood pressure regulation [41,42]. Nonetheless, BCAA effects on blood pressure may be counterbalanced in the presence of other nutrients: whey protein is rich in both BCAAs and lactokinins, the latter with inhibiting properties on the angiotensin-converting enzyme; thus, ingestion of whey protein may induce opposite effects on hypertension mediated by BCAAs and lactokinins [43]. Interestingly, AAAs and BCAAs have hydrophobic or bulky residues that can be relevant for the binding of bioactive peptides to the angiotensin-converting enzyme which is crucial in blood pressure control [44]. 

Concerning dietary glycine, findings from the INTERMAP study were not in line with other studies including glycine in factors obtained by principal component analysis [16,21,23].

In the INTERMAP study [21], dietary intake of glycine (expressed as a percentage of total protein and based on 24 h dietary recall) was positively associated with both systolic and diastolic blood pressure. Conversely, in a Greek study, plasma glycine levels—clustering with glutamine, serine, asparagine, threonine, lysine, histidine, and proline in the principal component analysis—showed a negative relationship with systolic blood pressure [16]. Also, in another study, the principal component analysis pattern encompassing glycine, sulfur amino acids, and alanine, tended to be associated to a diminished risk of hypertension [23]. An inverse correlation was observed between systolic blood pressure and sulfur amino acids in plasma, but not in cerebrospinal fluid, in a small Japanese cohort [17]. Stamler et al. [21] postulated that dietary glycine intake was positively associated with blood pressure, as glycine is abundant in animal-derived protein and meat consumption may be a dietary factor for elevated blood pressure. Conversely, the opposite findings may be supported by the important role played by glycine in reducing oxidative stress, favoring nitric oxide action; moreover, glycine is involved in the synthesis of structural protein, such as elastin; alterations in elastin formation have been connected to impaired elastic properties of vessels, a remarkable aspect in the pathogenesis of hypertension [45].

Dietary alanine showed a positive relation to blood pressure in the INTERMAP study (expressed as percentage of total protein intake) and in a nested cohort within the THIS-DIET study (as daily intake in absolute value) [21,25]. 

With respect to methionine, results based on dietary records [25] were in disagreement with findings from plasma amino acid levels [17]. Elevated dietary methionine intake was associated with augmented systolic and diastolic blood pressure [25]. Methionine is an essential amino acid; among its metabolic byproducts, homocysteine, when elevated, is well known for its ability to influence endothelial function inducing the production of asymmetrical dimethylarginine (ADMA), which in turn, can inhibit the synthesis of nitric oxide [46]. Thus, methionine effects on blood pressure appear to be indirect and mediated by the increase in homocysteine levels, as shown in studies relying on dietary supplementation with methionine in animals and humans [47,48]. 

Several amino acids interfere with vascular physiology; among them, arginine is well known to have relevant vasogenic properties [49]. Despite a wealth of studies demonstrating the beneficial effects from dietary supplementation with l-arginine including lowering both systolic and diastolic blood pressure levels [50,51], studies focusing on dietary arginine considering only a usual diet, excluding supraphysiological intake through dietary supplementation, did not support any association between this amino acid and blood pressure [11,18]. In middle-aged men participating in the Kuopio Ischaemic Heart Disease Risk Factor Study (KIHD), no association was found between dietary arginine and blood pressure levels, regardless of the dietary source (either plant-derived or animal-derived arginine) [11]. Similarly, dietary arginine did not associate with blood pressure in a Dutch elderly male population [18]. 

In the only study assessing urinary amino acids, a positive association was described between urinary cysteine, citrulline, and lysine and systolic blood pressure, and between urinary cysteine and diastolic blood pressure [13]. These data regarding urinary cysteine, as well as the lack of association for dietary cysteine and blood pressure [12] are in contrast with evidence showing antihypertensive effects mediated by cysteine through its free sulfhydryl group. The majority of studies on cysteine were based on supplementation with its stable analogue N-acetyl-cysteine [52]. Cysteine is able to modulate blood pressure by decreasing oxidative stress, increasing nitric oxide bioavailability, and ameliorating insulin sensitivity [52]. In addition, cysteine is part of the tripeptide glutathione with glutamic acid and glycine. Glutathione is in turn well known for its antioxidant ability with additional beneficial consequences on blood pressure regulation [52].

## 5. Conclusions

Due to the large variability in methodologies used for assessing amino acid levels and heterogeneity in results obtained, it was not possible to draw robust conclusions. In fact, the use of any type of dietary records was affected by under- or overreporting [53,54]. Data based on direct measurements in plasma and other biological fluids are more reliable, though they can be affected by derangements in metabolic cascades leading to alterations in amino acid metabolic byproducts. 

Further research should be prompted for a thorough understanding of the synergistic actions of different amino acid classes on blood pressure regulation. In addition, the interplay between gut microbiota and amino acid metabolism in hypertension [55] deserves future investigation. 

## Figures and Tables

**Table 1:** Study characteristics and main findings.

Authors	Year	Study Participants (*n*) and Study Design	Ethnicity	Age (Year) *	Amino Acids (Dietary or Biological Fluid Analysis)	Main Findings
Altorf-van der Kuil W et al. [12]	2013	3086 men and women (cross-sectional analysis) + 1810 men and women (prospective analysis, 6 year follow-up)	Dutch	66 ± 7	Glutamic acid, Arginine, cysteine, Lysine, Tyrosine (FFQ)	A higher intake of Tyr (∼0.3% of protein) was related to a 2.4 mm Hg lower SBP (*p*-trend = 0.05) but not to DBP (*p* = 0.35). The other AA were not significantly associated with BP levels in a cross-sectional analysis. None of the AA were associated with incident hypertension (HR: 0.81–1.18; *p*-trend> 0.2).
Cheng Y et al. [13]	2018	103 men and women DASH-Sodium trial—considering only the arm on a US diet (cross-sectional study)	US-American	49 ± 10	Urinary AAs	Significant positive associations with SBP for urinary cysteine (*p* = 0.008), Cit (*p* = 0.003), and Lys (*p* = 0.009); significant positive associations with DBP for urinary cystine (*p* = 0.005) but not the other metabolites.
Hu W et al. [14]	2016	1302 men and women Huai’an Diabetes Prevention Program (cross-sectional study)	Chinese	40–79	Serum BCAAs (Val, Ile, and Leu)	No significant correlation between either single BCAAs or total BCAAs, SBP, and DBP.
Jennings A et al. [15]	2016	1997 female twins Twin-UK cohort (cross-sectional study)	British	41.7 ± 12.1	Dietary BCAA (FFQ)	Higher BCAA intake was also associated with significantly lower SBP (Q5−Q1 = −2.3 mmHg, *p*-trend 0.01) comparing those in the highest and lowest quintiles of intake. Lower prevalence ratio for hypertension in the highest versus lowest quintile of BCAA intake (*p* = 0.02).
Ntzouvani A et al. [16]	2017	100 men (cross-sectional study)	Greek	54.6 ± 8.9	Five AA patterns by PCA: Factor 1: BCAAs and AAAs, glutamic and aspartic acid, Ala, Lys, and Met; Factor 2: Gln, Gly, Ser, Asn, Thr, Orn, Lys, His, and Pro; Factor 3: total homocysteine, cystathionine, creatinine, trimethyllysine, methylmalonic acid, Pro and kynurenine; Factor 4: betaine, choline, SDMA, dimethylglycine, and creatinine; Factor 5: Arg, TMAO, Orn and Met. (plasma)	Factor 2 was negatively associated with SBP (*r* = −0.296, *p* ≤ 0.005).
Ogawa M et al. [17]	1985	12 normotensive subjects and 12 patients with essential hypertension under nutritional control after at least 10 days of standard hospital diet (cross-sectional study)	Japanese	23–70	Plasma taurine, Ser, Met (sulfur AAs)(plasma and csf)	Plasma taurine, Ser, Met, and Thr were significantly lower in patients with essential hypertension than in normotensive patients. The levels of plasma taurine, Ser, Met, and total sulfur AAs correlated inversely to SBP. No difference was observed in the CSF levels of free AA in normotensive and hypertensive patients.
Oomen CM et al. [18]	2000	806 men The Zutphen Elderly Study (prospective cohort study, 10 year follow-up)	Dutch	64–84	Dietary arginine (cross-check dietary history method)	Non-significant lower SBP of approximately 2 mmHg with a 2.5 g/day higher Arg intake (*p* = 0.25).
Siomakajlo M et al. [19]	2017	263 men (cross-sectional study)	Polish	36–60	BCAAs, Phe, and AAAs: 2 factors by PCA: Factor 1: BCAAs—Leu, Ile, Val-, and Phe; Factor 2: Tyr and Trp (plasma)	Significant positive associations between Factor 1 and SBP (*r* = 0.15, 95% CI: 0.01; 0.3), and Factor 1 and DBP (*r* = 0.17, 95% CI: 0.03; 0.33).
Stamler J et al. [20]	2009	4680 men and women (cross-sectional study)	Chinese, Japanese, British, and US-American	40–59	Glutamic acid (24-h dietary recall)	Glutamic acid intake (as percentage of total protein) higher by 2 SD corresponded to lower DBP (z-score −2.15 to −3.57) and SBP (z-score −2.21 to −3.66).
Stamler J et al. [21]	2013	4680 men and women INTERMAP study (cross-sectional study)	Chinese, Japanese, British, and US-American	40–59	Glycine (24-h dietary recall)	Estimated average BP differences associated with a 2-SD higher Gly intake (0.71 g/24 h) were 2.0–3.0 mm Hg systolic BP (*z* = 2.97–4.32) stronger in Western than in East Asian participants.
Teymoori F et al. [22]	2019	4288 men and women (prospective analysis, 3.1 year follow-up)	Iranian	39.7 ± 12.8	Aromatic amino acids (FFQ)	The adjusted OR of hypertension for percentage of AAAs from total protein intake was 1.63 (95% CI, 1.06–2.50; *p* = 0.03) when comparing the highest quartile to the lowest. A positive relationship was observed between the highest versus the lowest quartile Phe intake (OR = 1.66; 95% CI, 1.14–2.47; *p* = 0.03). No significant association of Tyr and Trp intakes with hypertension risk.
Teymoori F et al. [23]	2017	4288 men and women (prospective analysis, 3.1 year follow-up)	Iranian	39.7 ± 12.8	Three AA patterns by PCA: 1st pattern: Branched chain AAs, aromatic AAs, and Pro. 2nd pattern: acidic AAs, and proline. 3rd pattern: sulfuric AAs, and small AAs (Gly and Ala) (FFQ)	The OR for incidence of hypertension of the highest quartile score of the first pattern was 1.83 (95% CI: 1.21–2.77, *p* for trend = 0.002), compared to the lowest. For the 2nd and 3rd patterns of dietary AA intake, no significant association with incident hypertension was found, although the 3rd pattern did have a slight tendency to reduce the risk of hypertension (OR = 0.81; 95% CI: 0.65–1.16, *p* for trend = 0.20).
Teymoori F et al. [24]	2018	4288 men and women (prospective analysis, 3.1 years follow-up)	Iranian	39.7 ± 12.8	AA ratios of Leu.Ser/Thr.Trp, Leu/Trp, Leu/Thr, and Ser/Thr (FFQ)	The OR of the highest quartile of dietary Leu.Ser/Thr.Trp intake was 1.48 (95% CI: 1.04–2.09, *p* for trend: 0.02), compared with the lowest one. The OR of hypertension in the highest, compared with the lowest quartile of the Leu/Thr ratio (2.19 versus 2.02) was 1.46 (1.01–2.12), *p* for trend = 0.07.
Tuttle KR et al. [25]	2012	92 men and women THIS-DIET study (nested cohort, prospective analysis, 2 year follow-up)	95% White	59 ± 9	Methionine, alanine, threonine, histidine. (3-day food diary)	Met and Ala were positively associated with higher SBP (OR (95% CI): 1.29 (1.14–1.46), *p* < 0.001, and 1.17 (1.05–1.30), *p* = 0.005) and higher DBP (OR (95% CI): 1.21 (1.05–1.39), *p* = 0.007, and 1.22 (1.07–1.38), *p* = 0.002). Thr and His were inversely associated with SBP (OR (95% CI): 0.84 (0.74–0.96), *p* = 0.01, and 0.92 (0.86–1.00), *p* = 0.04) and DBP (His only: OR (95% CI): 0.89 (0.82–0.97), *p* = 0.01)
Venho B et al. [11]	2002	1981 men The Kuopio Ischaemic Heart Disease Risk Factor Study (KIHD) (prospective cohort study, dietary arginine assessment just at baseline)	Finnish	52.3 ± 5.3	Total dietary arginine, animal-derived arginine, plant-derived arginine (4-day dietary record)	The regression coefficients between SBP and the intake of total, animal-derived and plant-derived Arg were 0.01 (*p* = 0.674), <0.01 (*p* = 0.931), and 0.02 (*p* = 0.420), respectively. The regression coefficients between DBP and the intake of total, animal-derived, and plant-derived Arg were <0.01 (*p* = 0.746), 0.01 (*p* = 0.831), and <0.03 (*p* = 0.195), respectively.
Yamaguchi N et al. [26]	2017	8589 men and women (cross-sectional study)	Japanese	>20	19 plasma free amino acids were measured: Ala, Arg, Asn, Cit, Gln, Gly, His, Ile, Leu, Lys, Met, Orn, Phe, Pro, Ser, Thr, Trp, Tyr, and Val	BCAAs and AAAs showed moderately positive correlation with SBP and DBP.
Yang R et al. [27]	2014	272 men and 200 women (cross-sectional study)	Chinese	70.1 ± 6.6	Serum BCAAs (Val, Ile, and Leu) and AAAs (Tyr and Phe)	Positive correlations between total AAAs and DBP (*r* = 0.127), and total BCAAs and DBP (*r* = 0.197) (all *p* < 0.05) as well as Val and SBP (*p* < 0.05). All single AAAs and single BCAA were positively correlated with DBP (all *p* < 0.05).

Legend: BP: blood pressure; CSF: cerebrospinal fluid; SBP: systolic blood pressure; DBP: diastolic blood pressure; DASH: Dietary Approaches to Stop Hypertension; AA: amino acid; AAAs: aromatic amino acids; BCAAs: branched chain amino acids; PCA: principal component analysis; FFQ: food frequency questionnaire; Ala: alanine; Arg: arginine; Asp: asparagine; Cit: citrulline; Gln: glutamine; Gly: glycine; His: histidine; Ile: isoleucine; Leu: leucine; Lys: lysine; Met: methionine; Orn: ornithine; Phe: phenylalanine; Pro: proline; Ser: serine; SDMA: symmetric dimethylarginine; Thr: threonine; TMAO: trimethylamine N-oxide; Trp: tryptophane; Tyr: tyrosine; Val: valine. SD: standard deviation; * Age expressed as mean ± SD, or age range when mean age of total participants was not clearly stated.
